# Perianal Skin Tags Revealing Asymptomatic Crohn’s Disease in a 12-Year-Old Child With Growth Impairment: A Case Report

**DOI:** 10.7759/cureus.37513

**Published:** 2023-04-12

**Authors:** Olga Bellou, Dimosthenis Chrysikos, Ourania Schoinohoriti, Ioannis Alexandrou, Efstratios Christianakis

**Affiliations:** 1 2nd Department of General Surgery, KAT General Hospital of Athens, Athens, GRC; 2 Department of Anatomy, National and Kapodistrian University of Athens, Athens, GRC; 3 Department of Oral and Maxillofacial Surgery, School of Dentistry, University of Athens, Athens, GRC; 4 Department of Pediatric Surgery, General Children Hospital of Penteli, Athens, GRC

**Keywords:** treatment, diagnosis, growth impairment, perianal skin tags, asymptomatic, pediatric crohn’s disease

## Abstract

Pediatric Crohn’s disease (CD) is a chronic inflammatory bowel disease considered to impair the growth of children and adolescents. Since CD commonly presents perianal manifestations, general surgeons may play a crucial role in its diagnosis and treatment. Detailed history, along with a thorough clinical examination, is mandatory for the management of CD perianal lesions. However, surgical intervention is only indicated in selected patients since it may lead to poor wound healing and recurrence. The article reports a case of a 12-year-old girl, presenting perianal skin tags and growth impairment as the first signs of asymptomatic CD.

## Introduction

Crohn's disease (CD) is a chronic transmural inflammatory disorder, potentially affecting any part of the GI tract from mouth to anus but frequently involving the perianal region. Its etiology is unknown, although genetic and environmental components have been inculpated, causing overactivation of T-cells and leading to tissue destruction [1]. Moreover, the pediatric CD has been designated as one of the diseases affecting the development of children and adolescents. Besides its typical symptoms (diarrhea, hemorrhagic stools, abdominal pain), CD is often associated with malnourishment, which may lead to growth impairment [2]. The perianal CD has been defined as inflammation at or near the anus, including tags, fissures, fistulae, abscesses, and stenosis, with an incidence reportedly varying from 3.8 to 80% in different populations [3]. Its symptoms include pain, itching, bleeding, purulent discharge, and stool incontinence. Perianal skin tags have been found to occur in up to 70% of the patients, sometimes signaling the diagnosis of CD months or even years before GI symptoms become apparent [4]. The differential diagnosis of perianal lesions in pediatric patients includes hemorrhoids, hemangiomas, rectal prolapse, CD, sexual abuse, condylomas, molluscum contagiosum, and various other conditions [3], causing significant anxiety and concern for their parents. In this report, we present the case of a 12-year-old female patient, presenting perianal skin tags as the first manifestation of pediatric CD, without any symptoms from the GI tract, along with growth impairment and small uterus, probably owed to a co-existing small pituitary gland.

## Case presentation

**Figure 1:**
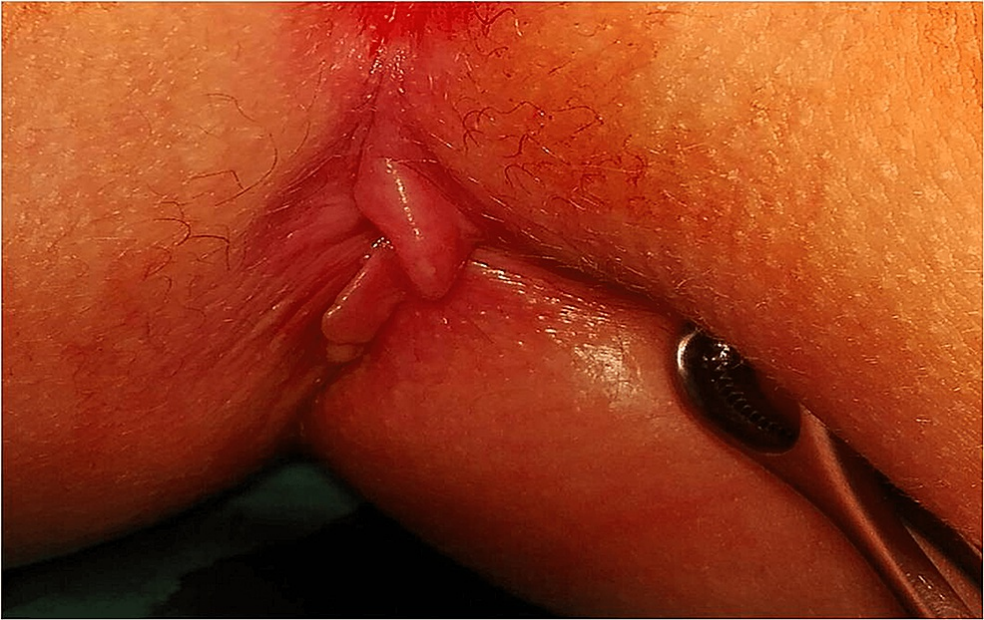
A 12-year-old female with perianal elephant-ear type skin tags masquerading as active Crohn's disease.

A 12-year-old girl with perianal skin tags was referred for examination to the Outpatient Clinic of the Pediatric Surgery Department at 'Penteli Children's Hospital of Athens,' Greece. Her medical history was unremarkable, without neither GI symptoms (pain, cramping, diarrhea, bleeding) nor fever, fatigue, reduced appetite, or weight loss, suggesting the presence of pediatric Crohn's disease (CD). Moreover, there were neither concerns for sexual abuse nor a history of human papillomavirus infection. Physical examination revealed three skin tags; one located at the 12th hour and two elephant ear-like tags located at the 6th hour of the anus (Figure 1).

**Figure 2:**
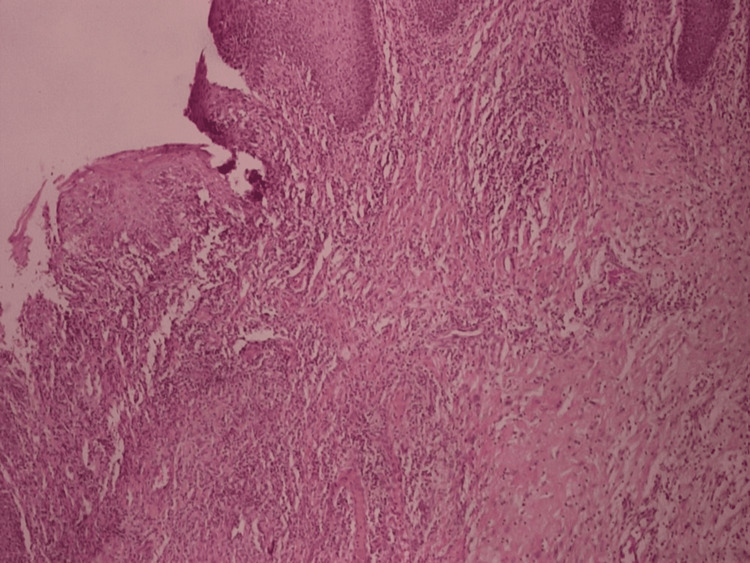
H&E staining of an anal mucosa specimen, excised from the ulceration site, showing considerable inflammatory infiltration of the stroma (magnification x 10).

The child, presenting a BMI of 19.23 kg/m2 (height 135 cm, weight 35 kg), was referred to the Developmental Pediatric Department, where she was diagnosed with a one-year delay in bone age. Laboratory values revealed microcytic anemia (hemoglobin 10.4 g/dL, hematocrit 32.6%, mean corpuscular volume 70.10 fl), thrombocytosis (platelet count 555.000/μlt), and remarkably low levels of insulin-like growth factor-1 (95.4 ng/ml with a normal range from 147 to 549 ng/ml). Immunologic tests (including IgA antibodies against tissue transglutaminase, anti-thyroglobulin antibodies, anti-thyroid peroxidase antibodies, anti-gliadin antibodies, and anti-endomysial antibodies) were within normal range. The 'elephant ear' skin tags at the 6th hour were excised for biopsy once active rectal inflammation was excluded through rectal examination. Anoscopy was performed under general anaesthesia before surgical excision of the tags, performed with a scalpel and followed by suturing, without intraoperative or postoperative bleeding. In 15 days, skin tags reappeared at the surgical site; antibiotic and anti-inflammatory drugs were prescribed, and complete remission was observed within a few days. The histology report revealed acanthosis, upper dermal edema, mild inflammatory infiltration, and vacuolar and lichen sclerosis-like changes compatible with CD (Figure 2).

Complete endoscopy and imaging of the GI tract were performed following the diagnosis of CD. More specifically, the patient was submitted to upper and lower GI endoscopy. Endoscopy revealed active CD perianally, within the stomach, duodenum, ileum, and rectosigmoid. The endoscopic features were non-specific, including a) diffuse gastroduodenal mucosal involvement with erythema and linear ulcers and b) "cobblestone appearance" and longitudinal ulcers in ileocolonoscopy. Magnetic resonance enterography revealed increased mural signal intensity with asymmetric bowel wall thickening and hyperenhancement, mostly along the mesenteric side of the ileum, findings consistent with active inflammation secondary to CD. An abdominal ultrasound showed no significant findings except for uterine developmental delay. The child was evaluated by endocrinologists for amenorrhea. Ophthalmological and dermatological testing did not reveal signs of systemic disease. Cerebral MRI demonstrated a small-sized pituitary gland without focal pathology. The patient was treated with high doses of corticosteroids (per os prednisone dose of 2 mg/kg/day for four weeks). Following the patient's clinical improvement (i.e., considerable size reduction of the skin tags upon physical examination), the daily corticosteroid dose was reduced slowly over an eight-week period until complete discontinuation. In addition, 3 mg/kg of azathioprine (AZA) was co-administered daily. After nine months of follow-up, the child remains asymptomatic without signs of active CD and total remission of the perianal skin tags. It is worth mentioning that the girl's growth has been restored to normal since she has gained 1 cm in height and 2 kg in weight.

## Discussion

Perianal skin tags are present in up to 70% of patients with perianal Crohn's disease (CD); their appearance may precede intestinal disease in months or years [4]. Skin tags, generally fleshy and occasionally pendulous in appearance, may be associated with fissures and are classified into two types. The first represents the typical CD skin tag and is usually large, edematous, hard, and cyanotic. These tags are attributed to lymphedema, owed to lymphatic obstruction, and often co-exist with intestinal inflammation. Their surgical excision is strongly contraindicated, as poor wound healing and/or recurrence may follow; thus, their management mainly consists in treating the underlying GI disease [4, 5]. The second type of tags are usually flat, broad or narrow, soft and painless, and are often described as 'elephant ears' [4, 5]. These may be excised as soon as active rectal inflammation has been excluded. In patients whose diagnosis of CD has not been corroborated, like in our case, an elephant ear-like skin tag should be submitted to excision biopsy since it has been shown to harbor granulomas, diagnostic for CD in up to 30% of patients [6]. The diagnostic approach should include a careful history, along with physical and endoscopic/anoscopic evaluation. These, together with a combination of magnetic resonance enterography or endoscopic ultrasound, depending on institutional expertise, provide the optimal diagnostic strategy. Moreover, it is essential to include the pediatric CD in the differential diagnosis of growth impairment in children and adolescents, as was discovered in our patient. Therefore, a multidisciplinary approach requiring assessment and treatment by various specialists (pediatricians, gastroenterologists, endocrinologists, and radiologists) is warranted. Azathioprine (AZA) in a daily dose of 3 mg/kg has been documented as a safe, well-tolerated, and effective maintenance therapy for children with moderate-to-severe inflammatory bowel disease [6, 7]. AZA is an immunomodulatory drug useful in treating CD thanks to its steroid-sparing effect, maintained remission, and decreased rates of hospitalization and surgery [6, 7].

## Conclusions

In conclusion, it is essential to recognize perianal skin tags in children as a sign of asymptomatic CD. Such lesions, leading to erroneous investigation for sexual abuse or other perianal disease, may cause unwarranted trauma for both the patient and his/her family. A multidisciplinary approach with assessment and treatment by various specialists is required in these patients since growth impairment may also be present in asymptomatic CD, as was noted in our patient. Although surgical evaluation of perianal skin tags is mandatory, extensive surgical intervention should be undertaken with caution due to the enhanced risk of poor wound healing and recurrence.
